# Association of growth hormone deficiency with an increased number of preadipocytes in subcutaneous fat

**DOI:** 10.3389/fendo.2023.1199589

**Published:** 2023-05-26

**Authors:** Lidan Zhao, Dan Jia, Zhendong Tan, Honglin Jiang

**Affiliations:** School of Animal Sciences, Virginia Tech, Blacksburg, VA, United States

**Keywords:** preadipocyte, adipogenesis, growth hormone, stromal vascular fraction, mice

## Abstract

The inhibitory effect of growth hormone (GH) on adipose tissue growth is well known, but the underlying mechanism is not fully understood. In this study, we determined the possibility that GH inhibits adipose tissue growth by inhibiting adipogenesis, the process of formation of adipocytes from stem cells, in the lit/lit mice. The lit/lit mice are GH deficient because of a spontaneous mutation to the GH releasing hormone receptor (ghrhr) gene, and they have more subcutaneous fat despite being smaller than the lit/+ mice at the same age. We found that cells of the stromal vascular fraction (SVF) of subcutaneous fat from the lit/lit mice had greater adipogenic potential than those from the lit/+ mice, as evidenced by forming greater numbers of lipid droplets-containing adipocytes and having greater expression of adipocyte marker genes during induced adipocyte differentiation in culture. However, addition of GH to the culture did not reverse the superior adipogenic potential of subcutaneous SVF from the lit/lit mice. Through florescence-activated cell sorting and quantification of mRNAs of preadipocyte markers, including CD34, CD29, Sca-1, CD24, Pref-1, and PPARγ, we found that subcutaneous SVF from the lit/lit mice contained more preadipocytes than that from the lit/+ mice. These results support the notion that GH inhibits adipose tissue growth in mice at least in part by inhibiting adipogenesis. Furthermore, these results suggest that GH inhibits adipogenesis in mice not by inhibiting the terminal differentiation of preadipocytes into adipocytes, rather by inhibiting the formation of preadipocytes from stem cells or the recruitment of stem cells to the fat depot.

## Introduction

The inhibitory effect of growth hormone (GH), also known as somatotropin, on adipose tissue growth has been demonstrated in different species. In rats, administration of pituitary GH reduced body fat while increasing lean body mass (1, 2). In obese mice, GH treatment reduced body fat mass in a dose-dependent manner (3). In GH receptor (GHR) null mice, fat mass percentage was markedly elevated (4). In pigs, administration of exogenous GH decreased body fat mass by as much as 70% (5, 6). In cattle, administration of GH from 0 to 160 mg/week decreased linearly the deposition of subcutaneous fat, visceral fat, and intramuscular fat (7). Maison et al. concluded that GH had significant fat mass-reducing effects in humans too after reviewing thirty-seven trials where GH treatment was given to adult patients with GH deficiency (8).

Adipose tissue grows *via* both hypertrophy and hyperplasia (9). Hypertrophy in adipose tissue is determined by the balance between lipogenesis, the process of triglyceride synthesis, and lipolysis, the process of triglyceride breakdown (10). Hyperplasia in adipose tissue is determined by proliferation of preadipocytes, the adipocyte precursor cells, terminal differentiation of preadipocytes to adipocytes, and apoptosis of preadipocytes and adipocytes (11). Increasing evidence suggests that GH inhibits adipose tissue growth by affecting more than one of these processes. Growth hormone has potent lipolytic effects in animals, although the underlying mechanism is not clear (12, 13). Earlier studies suggested that GH enhanced catecholamine-stimulated lipolysis (14–17), but more recent studies suggested that GH stimulates lipolysis by activating peroxisome proliferator activated receptor γ (PPARγ), which then inhibits the expression of fat-specific protein 27 (FSP27) in adipose tissue (18, 19). Growth hormone reduces fat mass also by suppressing lipogenesis. The inhibitory effect of GH on lipogenesis has been well demonstrated in pigs (20–25). Mechanistically, GH inhibits lipogenesis by antagonizing the stimulatory effect of insulin on lipogenesis (26).

In theory, GH could inhibit adipose tissue growth by inhibiting adipogenesis, the process of formation of adipocytes (27, 28). However, studies on this possibility have generated seemingly conflicting results. Whereas studies in primary preadipocyte culture indicated an inhibitory effect of GH on preadipocyte differentiation into adipocytes (29–31), studies in preadipocyte cell lines, such as 3T3-L1, 3T3-F442A, and Ob1771, revealed a stimulatory effect of GH on this process (32–36). These controversies beg additional studies to clarify the effect of GH on adipogenesis.

In this study, we determined the effect of GH on adipogenesis in the GH-deficient lit/lit mice. The lit/lit mice carry a mutation that disable the GH releasing hormone receptor (*ghrhr*) gene, and as such, mice homozygous for the mutation (lit/lit) are deficient in GH and smaller than wild-type or lit/+ mice (37, 38). In a previous study, we found that the lit/lit mice had more subcutaneous fat than the lit/+ mice despite being much smaller than the latter (39). In this study, we found that the lit/lit mice accumulated more preadipocytes in subcutaneous fat but that terminal differentiation of preadipocytes from the lit/lit mice into adipocytes was not affected by GH *in vitro*.

## Materials and methods

### Mice and adipose tissue collection

Breeding pairs of C57BL/6J-Ghrhr^lit^ mice, a lit/lit female and a lit/+ male, were purchased from The Jackson Laboratory (Bar Harbor, ME, USA). Mice were housed on an automatically timed 12 h light/dark cycle at 23°C with free access to standard rodent chow and water. Male lit/+ littermates were used as controls for lit/lit mice because wild-type littermates were not available from the breeding program and because lit/+ mice did not differ from wild-type mice in body weight (38). Mice were euthanized by CO_2_ inhalation and cervical dislocation. Euthanasia was performed between 9:00 a.m. and 11:00 a.m. Mice were euthanized for collection of the inguinal fat pads. The inguinal fat pads in this study referred to the subcutaneous fat pads between the lumbar spine and the groin. All animal-related procedures were approved by the Virginia Tech Institutional Animal Care and Use Committee.

### Isolation and culture of stromal vascular fraction

The stromal vascular fraction (SFV) of adipose tissue was isolated as previously described with minor modifications (40). Fat pads were minced and digested using 1 mg/ml of collagenase D (Roche, Indianapolis, IN, USA) in HEPES buffer (100 mM HEPES pH 7.4, 120 mM NaCl, 50 mM KCl, 0.5 mM glucose, 1.5% bovine serum albumin, and 1 mM CaCl_2_) at 37 °C with shaking at 115 rpm for 1 h. After centrifugation at 500 ×g for 10 min at room temperature, the pelleted SVF was washed twice with growth medium consisting of Dulbecco’s Modified Eagle’s Medium (DMEM)/F12 (Sigma-Aldrich, St. Louis, MO, USA), 10% fetal bovine serum or FBS (Atlanta Biologicals, Lawrenceville, GA, USA), 2.5 mM of L-glutamine, and 1% of antibiotics-antimycotics or ABAM (Mediatech, Manassas, VA, USA).

The SVF cells were initially cultured in growth medium in a CO2 incubator at 37 °C. Four or 5 days later, the SVF cells from the same mouse were collected and reseeded in 6- or 24-well plates at a density of 25,000/cm^2^. When cells reached 100% confluence, they were induced to differentiate into adipocytes using the previously described method (40). This method included initially culturing the cells in the DMEM/F12 medium supplemented with 5% FBS, 17 nM insulin (Sigma-Aldrich), 0.1 μM dexamethasone (Sigma-Aldrich), 250 μM 3-isobutyo-1-methylxanthine (IBMX) (Sigma-Aldrich), and 60 μM indomethacin (MP Biomedical, Solon, OH, USA) for two days, then in DMEM/F12 medium supplemented with 17 nM insulin and 10% FBS for another two days, and lastly in DMEM/F12 supplemented with 10% FBS for four days. Each cell culture experiment was repeated 3 or 4 times, each time using cells isolated from different lit/lit or lit/+ mice.

### Oil Red O staining

Cells were washed with phosphate buffered saline (PBS) twice and fixed with 10% phosphate buffered formalin (Fisher Scientific, Pittsburg, PA, USA) for 1 h. Cells were washed first with water and then 60% isopropanol (Fisher Scientific). Cells were stained with 2.1 mg/ml Oil Red O solution prepared in 60% isopropanol for 1 h and then washed with water as previously described (41).

### RNA isolation and real-time PCR

Total RNA from cultured adipocytes was isolated using TRI reagent (Invitrogen, Grand island, NY, USA) according to the manufacture’s instruction. Total RNA (1 μg/reaction) was reverse-transcribed into cDNA using ImProm-II Reverse Transcription system (Promega, Madison, Wisconsin, USA). cDNA (20 ng/reaction) was quantified by PCR using gene-specific primers (**Table 1**) and Fast SYBRGreen Master Mix on an Applied Biosystems 7500 Real-Time PCR machine (Applied Biosystems, Foster City, CA, USA). Specificity of primers was validated by gel electrophoresis and DNA sequencing of PCR products, and efficiency of primers was validated by amplifying a serial dilution of cDNA. Each sample was quantified in duplicate. The PCR data were analyzed using the 2(-ΔΔCt) method (42). The mouse *hmbs* gene was chosen as the reference gene because, based on the Ct values, its mRNA expression was stable across the samples.

**Table 1:** PCR primers used in this study.

Gene	Primer sequence (5’ to 3’)	GenBank #	Amplicon size (bp)
*pparg*	Forward: TTCAGAAGTGCCTTGCTGTG	NM_001127330.1	84
	Reverse: CCAACAGCTTCTCCTTCTCG		
*cebpa*	Forward: AGCAACGAGTACCGGGTACG	NM_007678.3	71
	Reverse: TGTTTGGCTTTATCTCGGCTC		
*lipe*	Forward: TCGCTGTTCCTCAGAGACCT	NM_001039507	151
	Reverse: CTGCCTCAGACACACTCCTG		
*plin1*	Forward **:** AAGGATCCTGCACCTCACAC	NM_175640.2	191
	Reverse: CCTCTGCTGAAGGGTTATCG		
*cd24*	Forward: ATGCCGCTATTGAATCTGCTGGAG	NM_009846	210
	Reverse: TGCACTATGGCCTTATCGGTCAGA		
*itgb1*	Forward: CAATGGCGTGTGCAGGTGTC	NM_010578.2	207
	Reverse: ACGCCAAGGCAGGTCTGAC		
*cd34*	Forward: GCAGGTCCACAGGGACACGC	NM_001111059.1	198
	Reverse: TGGCTGGTACTTCCAGGGATGCT		
*ly6a*	Forward: GGGACTGGAGTGTTACCAGTGCTA	NM_010738.2	166
	Reverse: AGGAGGGCAGATGGGTAAGCAA		
*dlk1*	Forward: GCGTGGACCTGGAGAAAGGCCA	NM_010052.5	276
	Reverse: GGAAGTCACCCCCGATGTCGGT		
*hbms*	Forward: AGAAAAGTGCCGTGGGAACC	NM_013551.2	107
	Reverse: GAGGCGGGTGTTGAGGTTT		

### Fluorescence-activated cell sorting

Preadipocytes from the mouse subcutaneous SVF were sorted and counted as previously described (43). To describe in brief, freshly isolated SVF cells were resuspended in ice-cold DMEM with 2% FBS and then incubated with the following antibodies at 1:100 dilution on ice for 15 min: APC-Cy7 rat anti-mouse CD45 (Cat# 557659, RRID : AB_396774), PE-Cy7 rat anti-mouse CD31 (Cat# 561410, RRID : AB_10612003), PerCP-Cy5.5 rat anti-mouse TER-119/erythroid cells (Cat# 560512, RRID : AB_10561844), PE hamster anti-rat CD29 (Cat# 562154, RRID : AB_10897843), BV421 rat anti-mouse CD34 (Cat# 562608, RRID : AB_11154576), PE-CF594 rat anti-mouse Ly-6A/E (also known as Sca-1) (Cat# 562730, RRID : AB_2737751), and APC rat anti-mouse CD24 (Cat# 562349, RRID : AB_11151896), all from BD Biosciences (San Jose, CA, USA). Following the incubation, cells were washed with Hank’s Balanced Salt Solution (HBSS) and then resuspended in HBSS supplemented with 0.5 g/ml propidium iodide (BD Biosciences). Cells were subsequently sorted on a BD FACSARIA Fusion Flow Cytometer equipped with BD FACSDiva Software (BD Biosciences). Cells were sorted first for singlets, followed by exclusion of propidium iodide-stained cells, i.e., dead cells. Live single cells were then sorted on the basis of cell-surface markers CD45, CD31, and TER-119. Cells negative for these markers (i.e., lineage negative or LIN−) were then sorted based on their expression of CD29, CD34, Sca-1, and CD24, the cell surface markers for preadipocytes (43).

### Statistical analyses

Data from multiple groups were analyzed by ANOVA followed by Tukey’s test. Data from two groups were analyzed by *t*-test. These analyses were performed using the General Linear Model in JMP (SAS Institute Inc., Cary, NC, USA). The experimental units for all experiments in this study were mice. All data are expressed as the mean ± standard error (SE).

## Results

### Stromal vascular fractions from the lit/lit mice had greater adipogenic potential than those from the lit/+ mice

**Figure 1:**
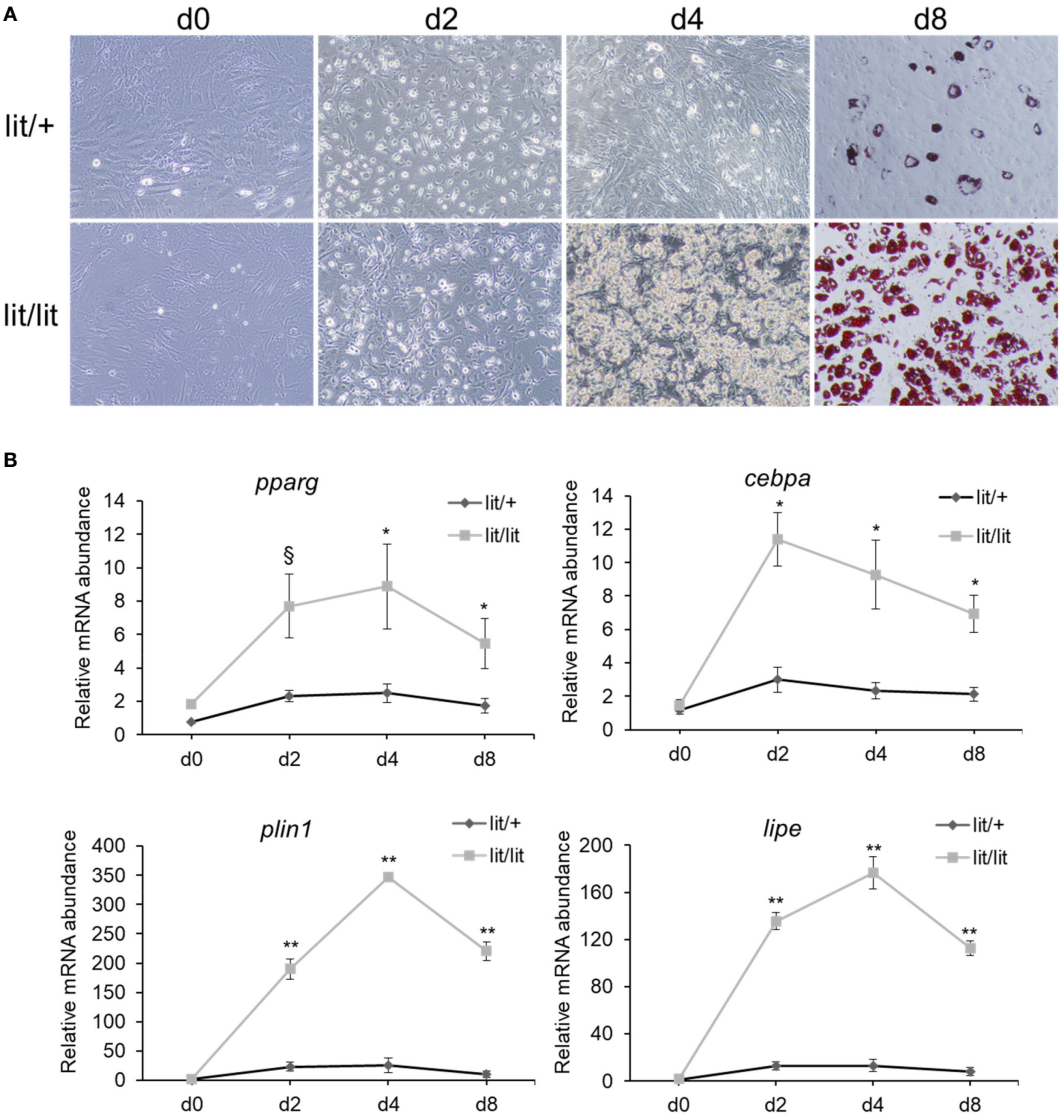
Differentiation of stromal vascular fraction (SVF) of subcutaneous fat from lit/+ and lit/lit mice to adipocytes. Stromal vascular fractions were isolated from inguinal subcutaneous fat pads of 13-week-old mice and were induced to differentiate to adipocytes in culture using a standard protocol (see text for details). This cell culture experiment was repeated 4 times, each time using SVF from a pair of lit/lit and lit/+ male mice. **(A)** Representative images of cells. Microscopic pictures were taken on day 0 (d0), d2, d4, and d8 of differentiation at 10x magnification. Cells on d8 of differentiation were stained with Oil Red O. **(B)** Expression levels of *pparg*, c*ebpa*, *plin1*, and *lipe* mRNAs in differentiating SVF cells. Relative abundance of mRNA was quantified by RT-qPCR. *Cebpa*, C/EBPα; *pparg*, PPARγ; *plin1*, perilipin-1; *lipe*, lipase e, hormone sensitive type, also known as hormone sensitive lipase. Data are expressed as means ± SE (n = 4 mice). mRNA expression was significantly different between days for both lit/lit and lit/+ (*P* < 0.01). *P < 0.05, **P < 0.01, and §*P* < 0.1 lit/lit *vs*. lit/+.

The subcutaneous fat SVFs isolated from 13-week-old male lit/+ and lit/lit mice were induced to differentiate into adipocytes in culture. During the 8-day course of differentiation induction, the SVF cells from both the lit/lit and lit/+ mice underwent similar morphological changes: they both changed from the fibroblast-like shape to spherical shape, and they gradually accumulated lipid droplets (**Figure 1A**). However, it was apparent that a much greater portion of the SVF cells from the lit/lit mice became lipid droplets-containing adipocytes than that from the lit/+ mice at days 2 and 4 of differentiation (**Figure 1A**), and these differences were confirmed by Oil Red O staining of cells at day 8 of differentiation (**Figure 1A**).

To further assess the adipogenic potential of the subcutaneous fat SVF cells from the lit/lit and lit/+ mice, mRNAs of four adipocyte marker genes were quantified. These marker genes were peroxisome proliferator activated receptor γ (*pparg*), CCAAT/enhancer-binding protein α (*cebpa*), perlipin-1 (*plin1)*, and hormone sensitive lipase (*lipe*). Genes *pparg* and c*ebpa* are two master transcriptional regulators of adipogenesis (27); genes *plin1* and *lipe* play an important role in lipid metabolism in adipocytes (44). At days 2, 4, and 8 of differentiation, almost all of these marker genes were expressed at much higher levels in the lit/lit than lit/+ SVF cells (*P* < 0.05, **Figure 1B**). These differences further indicated that the subcutaneous fat SVF cells from the lit/lit mice had much greater potential than those from the lit/+ mice at the same age to differentiate into adipocytes.

### Growth hormone had no effect on the potential of the lit/lit SVF to differentiate into adipocytes in vitro

**Figure 2:**
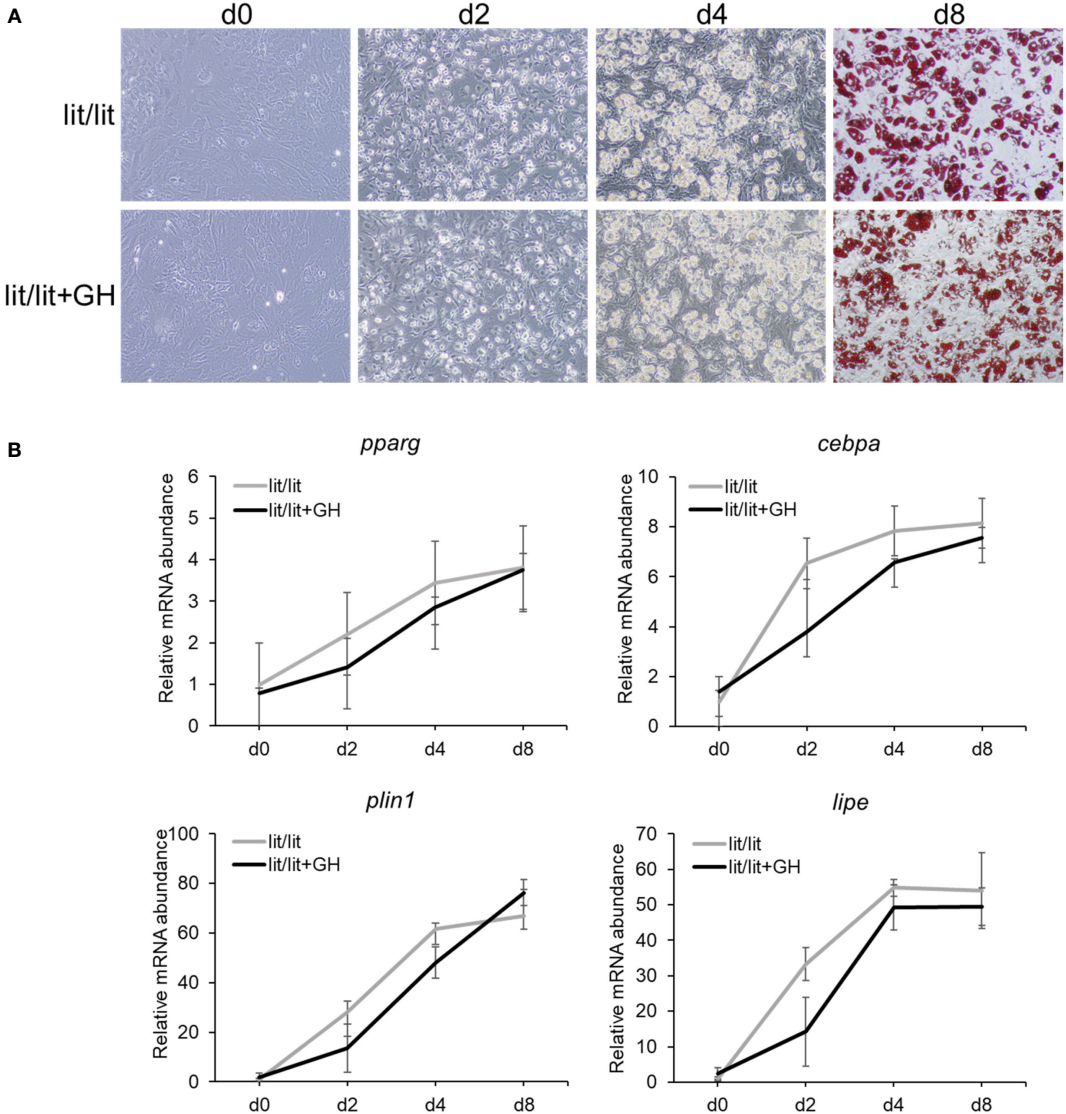
Effect of growth hormone (GH) on differentiation of stromal vascular fraction (SVF) from lit/lit mice to adipocytes. Stromal vascular fractions were isolated from inguinal subcutaneous fat pads of 14-week-old male lit/lit mice. Cells were induced to differentiate to adipocytes in the presence or absence of 100 ng/ml of recombinant bovine GH. This cell culture experiment was repeated 3 times, each time using SVF from a different mouse. **(A)** Representative pictures of cells. Microscopic pictures were taken on day 0 (d0), d2, d4, and d8 of adipocyte differentiation at 10x magnification. Cells on d8 of differentiation were stained with Oil red O. Note that there was no apparent difference in the formation of adipocytes between lit/lit and lit/lit+GH SVF. **(B)** Expression levels of *pparg*, c*ebpa*, *lipe* and *plin1* mRNAs in differentiating SVF cells. Data are expressed as means ± SE (n = 3 mice). The expression of these genes in both groups increased (*P* < 0.03) during adipocyte differentiation; however, the expression of these genes was not different (*P* > 0.8) between the two groups on the same days of differentiation.

The aforementioned data that the subcutaneous fat SVF cells from the GH-deficient lit/lit mice had greater adipogenic potential than those from the GH-normal lit/+ mice suggested that GH inhibited subcutaneous fat adipogenesis in mice. To further determine the effect of GH on adipogenesis, we differentiated the subcutaneous fat SVF from the lit/lit mice into adipocytes in the presence of 100 ng/ml bovine GH, which was previously shown to be effective in stimulating liver IGF-I gene expression or inhibiting adipose tissue growth in mice (45, 46). Surprisingly, based on the formation of lipid droplets-containing adipocytes (**Figure 2A**) and the expression levels of adipocyte marker genes (**Figure 2B**), GH addition had no significant effect on the differentiation of the lit/lit SVF cells into adipocytes in culture.

### The SVF from the lit/lit mice contained more preadipocytes than that from the lit/+ mice

**Figure 3:**
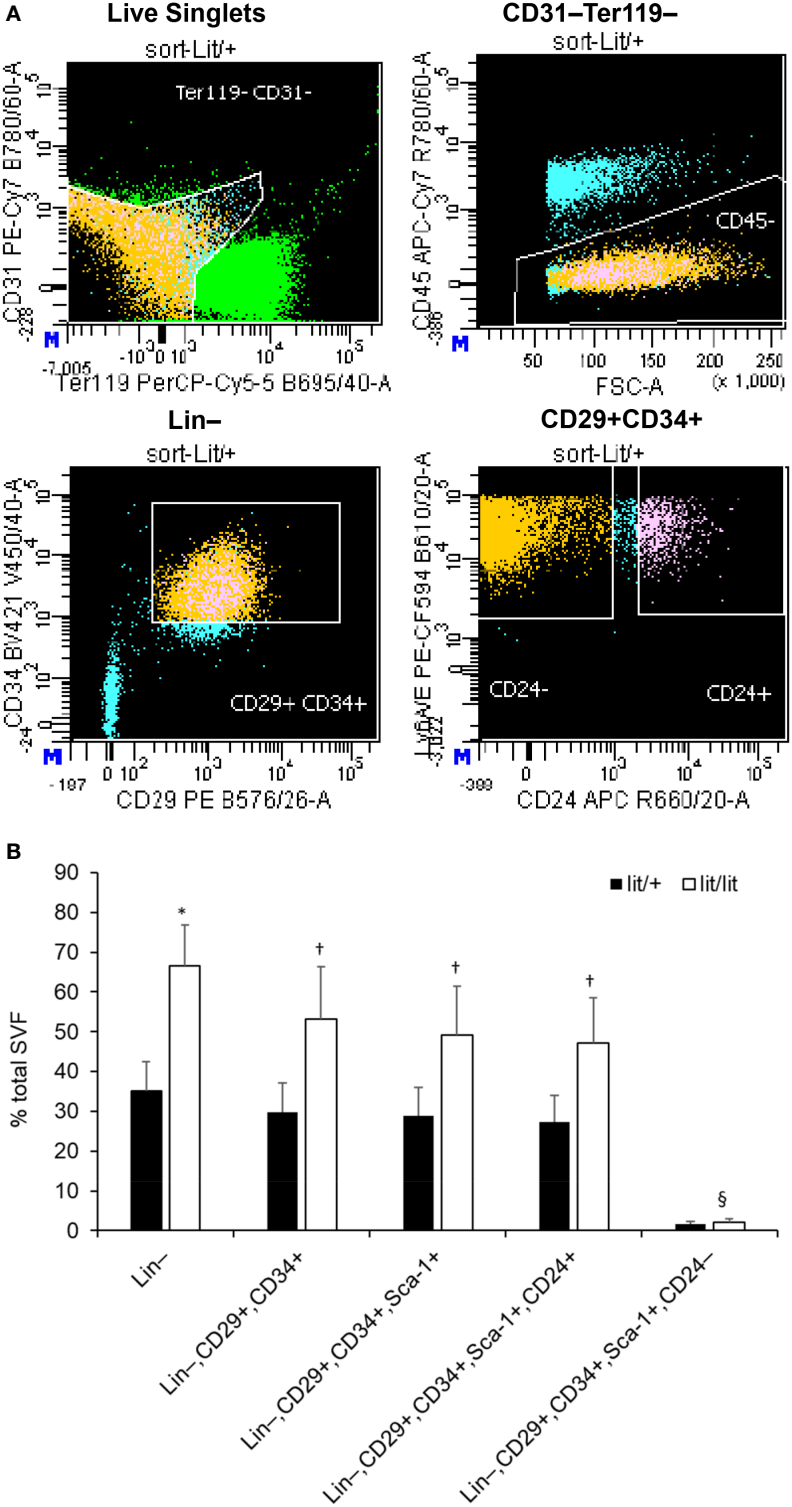
Fluorescence-activated cell sorting (FACS) of subcutaneous adipose SVF from lit/+ and lit/lit mice. The SVFs were isolated from 3 16-week-old male lit/+ and 3 16-week-old male lit/lit mice. **(A)** Representative FACS staining profiles and gating (white lines) of subcutaneous adipose SVF from a lit/lit mouse. The propidium iodide (PI)-negative singlets, i.e., live singlets, were sorted first for lack of Ter119 and CD31 and then lack of CD45 on cell surface. Ter119−, CD31−, and CD45− cells (i.e., Lin− cells) were then sorted on the basis of expression of CD34 and CD29. CD34+ and CD29+ cells were further sorted for expression of Sca-1 and CD24. **(B)** The percentages of total live single SVF cells expressing the indicated markers. Results are shown as mean ± SE (n = 3 mice). *P = 0.06, †*P* = 0.14-0.18, and §*P* = 0.73 *vs*. lit/+.

The SVF consists of several other types of cells in addition to preadipocytes (47). The aforementioned results that the SVF cells from the GH-deficient lit/lit mice had greater potential to become adipocytes than those from the GH-normal lit/+ mice yet that GH had no effect on the differentiation of the lit/lit SVF cells into adipocytes *in vitro* suggested that the lit/lit subcutaneous fat SVF might contain more preadipocytes, the adipocyte precursor cells, than the lit/+ subcutaneous fat SVF. To test this possibility, we first used FACS to compare the percentage of preadipocytes between the lit/lit and lit/+ SVFs. As shown in **Figure 3A**, we sorted and counted preadipocytes in the SVF based on their expression of surface markers CD29, CD34, Sca-1, and CD24 (43). The SVF from the lit/lit mice contained more Ter119–, CD31–, and CD45– cells, i.e., Lin– cells, than the SVF from the lit/+ mice (*P* < 0.05, **Figure 3B**). The SVF from the lit/lit mice had the tendency (*P* < 0.18, n = 3) to contain more Lin–, CD34+, CD29+, Sca-1+, and CD24+ cells, i.e., preadipocytes, than the SVF from the lit/+ mice (**Figure 3B**). CD24− preadipocytes are considered more committed toward becoming adipocytes than CD24+ preadipocytes (43, 48). The percentage of Lin–, CD29+, CD34+, Sca-1+, and CD24– cells, i.e., the more committed preadipocytes, was not different between the lit/lit and lit/+ SVF (**Figure 3B**).

**Figure 4:**
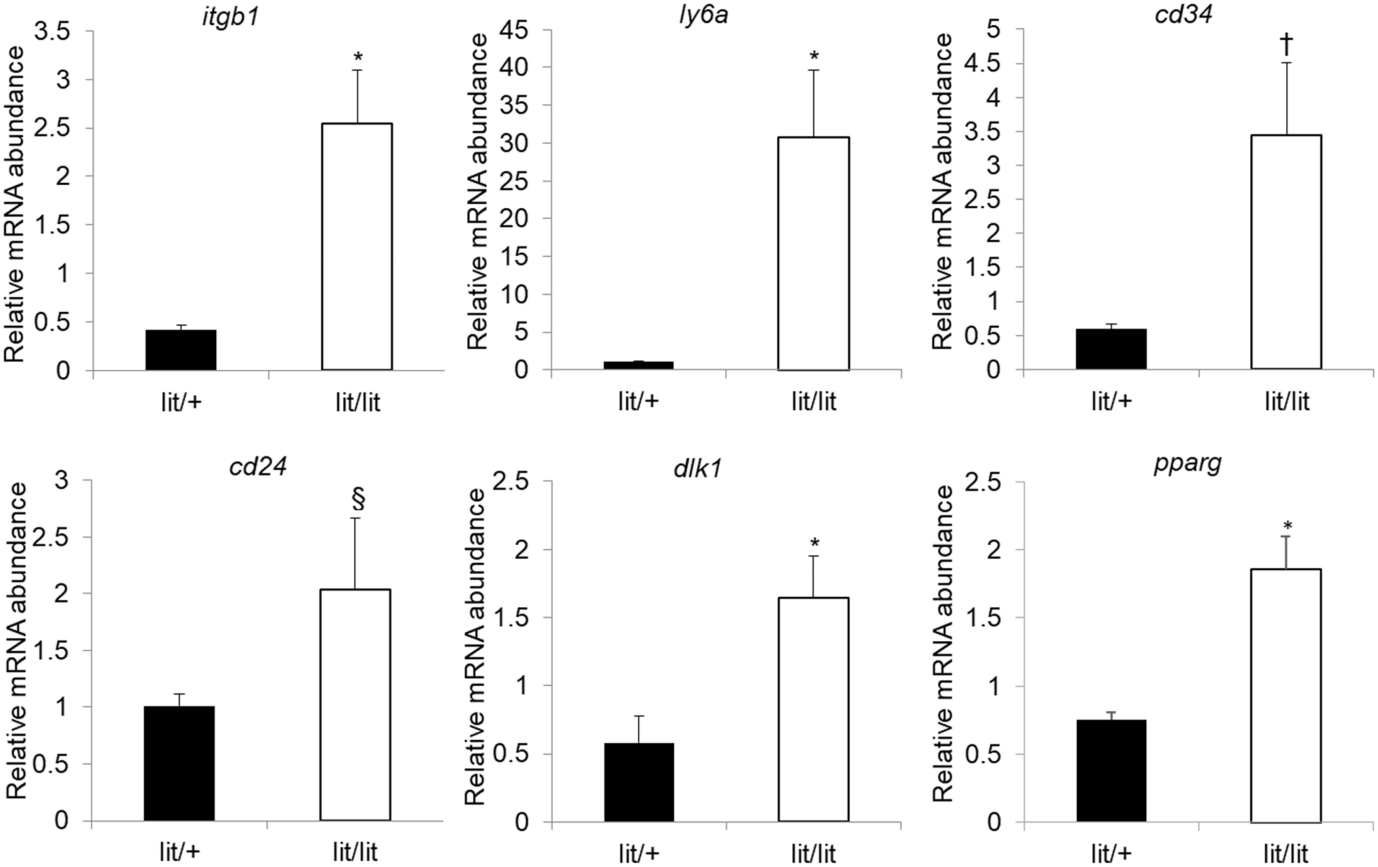
Quantification of mRNA levels of preadipocyte markers in subcutaneous adipose SVF cells. Stromal vascular fractions were isolated from inguinal subcutaneous fat pads of 16-week-old male lit/+ and lit/lit mice. mRNA expression was quantified by RT-qPCR. Data are expressed as means ± SE (n = 3 and 4 mice for lit/+ and lit/lit, respectively). *P < 0.05, †*P* = 0.07, and §*P* = 0.23 *vs*. lit/+*. Dlk1, itgb1, ly6a*, and *pparg* are also known as Pref-1, CD29, Sca-1, and PPARγ, respectively.

We also quantified the expression levels of *cd29*, *cd24*, *itgb1, ly6a, dlk1*, and *pparg* mRNAs in the subcutaneous fat SVF cells from the lit/lit and lit/+ mice. *Itgb1, ly6a, dlk1*, and *pparg* are genes encoding CD29, Sca-1, Pref-1, and PPARγ, respectively. Besides CD29, CD34, Sca-1, and CD24, which are cell surface proteins (43), Pref-1, a transmembrane protein and PPARγ, a nuclear protein, are also considered markers of preadipocytes (49, 50). In particular, Pref-1 is considered an excellent preadipocyte marker because it is highly expressed in preadipocytes but silenced in adipocytes (51). As shown in **Figure 4**, *cd29*, *itgb1, ly6a, dlk1*, and *pparg* mRNAs were all expressed at higher levels in the lit/lit than in the lit/+ SVF cells (*P* < 0.05); *cd24* mRNA expression had a tendency to be higher in the lit/lit than the lit/+ SVF cells (*P* < 0.1, n = 4). These gene expression data further indicated that the subcutaneous fat SVF from the GH-deficient lit/lit mouse contained more preadipocytes than that from the GH-normal lit/+ mouse.

## Discussion

The inhibitory effect of GH on adipose tissue growth has been demonstrated by multiple studies (4, 52, 53), but the underlying mechanism is not fully understood. In a previous study, we found that the GH-deficient lit/lit mice were growth retarded overall but accumulated more subcutaneous fat compared to the GH-normal lit/+ mice at the same age (39). These phenotypes of the GH-deficient lit/lit mice are consistent with those of GHR-null mice (54) and similar to the body weight and body composition changes in GH-deficient humans (53, 55). In this study, we aimed to gain new understanding of this mechanism by studying the effect of GH deficiency on adipose tissue growth in the lit/lit mice.

During the development of obesity, hypertrophy is considered to be the initial event (56). However, the existing adipocytes cannot enlarge infinitely; therefore, continuing increase of adipose tissue mass depends on the formation of new adipocytes from preadipocytes. Preadipocytes are the adipocyte precursor cells of adipose tissue SVF (49). We compared the differentiation potential of subcutaneous fat SVF cells from the lit/lit and age-matched lit/+ mice in vitro. Our experiments showed that the SVF cells from the lit/lit mice had greater potential to differentiate into adipocytes than those from the lit/+ mice, implying that GH inhibits adipogenesis in vivo. Similar implications have been inferred from studying SVF from the GHR-null mice and bovine GH-transgenic mice (57). Increased adipogenic potential of the lit/lit SVF could be attributable to the SVF containing more preadipocytes, or the existing preadipocytes being more sensitive to adipogenic hormones. However, we found that the increased ability of the lit/lit SVF cells to differentiate into adipocytes in response to a standard adipogenic cocktail was not reversed by GH at a supraphysiological concentration (100 ng/ml) in vitro. This result argues against the possibility that GH inhibits adipogenesis in mice by directly inhibiting the sensitivity of existing preadipocytes to adipogenic hormones. This result, however, does not rule out the possibility that GH inhibits differentiation of preadipocytes into adipocytes in vivo through an indirect mechanism.

According to Rodeheffer and associates (43), preadipocytes in mouse white fat bear the following surface markers: Lin−, CD29+, CD34+, Sca-1+, and CD24+. We used FACS to count preadipocytes in subcutaneous fat SVF from the lit/lit and lit/+ mice using antibodies against these markers and quantified the mRNA levels of these markers in these cells. In addition, we measured the mRNA levels of Pref-1 and PPARγ, two widely accepted markers of preadipocytes (49–51). The data from both FACS and mRNA quantification indicated that SVF from the lit/lit subcutaneous fat contained more preadipocytes than that from the lit/+ subcutaneous fat. Preadipocytes in a fat depot could come from increased commitment of stem cells toward preadipocytes in the depot, increased recruitment of stem cells to the depot, or increased proliferation of preadipocytes in the depot. A previous study in C3H10T1/2 cells, which are pluripotent mesenchymal stem cells, supports the possibility that GH inhibits the commitment of stem cells to becoming preadipocytes (58). As a growth stimulating hormone, it is unlikely that GH somehow inhibits preadipocyte proliferation. It remains to be determined if GH also inhibits the recruitment of stem cells to the subcutaneous fat depot in mice.

It should be pointed out that this study is not without limitations. One limitation of this study is that only 3 or 4 mice per group were used, and some of the observations would be more rigorous if larger sample sizes were used. A second limitation of this study was that we did not investigate the mechanism by which GH inhibits the recruitment of stem cells to the subcutaneous fat depot or the commitment of recruited stem cells to preadipocytes. BMP2 and BMP4 have been shown to be essential for the commitment of mesenchymal stem cells to the adipocyte lineage (28); so, it is possible that GH inhibits the conversion of mesenchymal stem cells to preadipocytes by inhibiting BMP2 or BMP4 gene expression or their signaling. The Wnt/beta-catenin pathway is known to suppress the commitment of mesenchymal stem cells to the adipocyte lineage (28). A previous study showed that overexpression of bovine GH gene in mice was associated with increased expression of Axin2, a key mediator of Wnt/beta-catenin signaling (57); therefore, GH might inhibit the commitment of mesenchymal stem cells to preadipocytes by enhancing Wnt/beta-catenin signaling.

**Figure 5:**
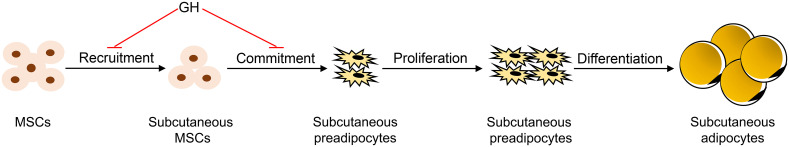
Model of how growth hormone (GH) inhibits subcutaneous adipogenesis in mice. It is hypothesized that GH inhibits the formation of adipocytes in mice by inhibiting the recruitment of mesenchymal stem cells (MSCs) to the subcutaneous fat depot, or the commitment of the multipotent MSCs to preadipocytes in the subcutaneous fat depot, or both. In this model, GH does not affect preadipocyte proliferation or terminal differentiation of preadipocytes into adipocytes.

In summary, the results of this study support the conclusions that GH inhibits subcutaneous fat growth in mice in part by inhibiting adipogenesis and that GH inhibits the formation of adipocytes in subcutaneous fat in mice by inhibiting the accumulation of preadipocytes in the subcutaneous fat depot (**Figure 5**). We propose that GH inhibits the accumulation of preadipocytes in subcutaneous fat by either inhibiting the recruitment of mesenchymal stem cells to the subcutaneous fat depot or inhibiting the commitment of mesenchymal stem cells to preadipocytes in the subcutaneous fat depot (**Figure 5**).

## Data availability statement

The raw data supporting the conclusions of this article will be made available by the authors, without undue reservation.

## Ethics statement

The animal study was reviewed and approved by Virginia Tech IACUC.

## Author contributions

HJ conceived the study; LZ and HJ designed the experiments; LZ, DJ, and ZT conducted the experiments; LZ, DJ, and ZT analyzed the data; LZ and HJ wrote the manuscript. All authors contributed to the article and approved the submitted version.
